# Human papillomavirus in high- and low-risk areas of oesophageal squamous cell carcinoma in China

**DOI:** 10.1038/sj.bjc.6603765

**Published:** 2007-04-24

**Authors:** K Shuyama, A Castillo, F Aguayo, Q Sun, N Khan, C Koriyama, S Akiba

**Affiliations:** 1 Department of Epidemiology and Preventive Medicine, Kagoshima University Graduate School of Medical and Dental Sciences, 8-35-1 Sakuragaoka, Kagoshima 890-8544, Japan; 2 Division of Radiation Epidemiology, National Institute for Radiological Protection, Chinese Center for Disease Control and Prevention, 2 Xinkang Street, Deshengmenwai, Xicheng District, Beijing 100088, China

**Keywords:** HPV, oesophageal squamous cell carcinoma, viral load, integration, China

## Abstract

To examine the potential roles of human papillomavirus (HPV) in oesophageal squamous cell carcinoma (ESCC) development, we examined the presence of HPV DNA in paraffin-embedded ESCC tissues collected from two areas with different ESCC incidence rates in China, that is, Gansu (*n*=26) and Shandong (*n*=33), using PCR with SPF10 primers, or PCR with GP5+/GP6+ primers combined with Southern blot hybridisation. HPV genotype was determined by the INNO-LiPA HPV genotyping kit. HPV DNA was detected in 17 cases (65%) in Gansu, where ESCC incidence is much higher than in Shandong, where HPV was positive in two samples (6%). HPV genotypes 16 and 18 were detected in 79 and 16% of HPV-positive samples, respectively. Real-time PCR analysis suggested the presence of integrated form of HPV DNA in all the HPV-16-positive samples, but its viral load was estimated to be only <1–2 copies cell^−1^. We could not detect HPV 16/18 E6 protein expression by immunostaining in any of the HPV-16-positive samples. Neither p16^INK4a^ nor p53 expression was related to HPV presence in ESCCs. Further studies seem warranted to examine the possible aetiological roles of HPV in ESCC.

Human papillomavirus (HPV) is a nonenveloped, double-stranded DNA virus with more than 90 genotypes. To date, molecular and epidemiological studies have convincingly demonstrated that HPV infection with certain genotypes, that is high-risk HPV, plays an essential role in the development of uterine cervical cancer (Munoz *et al*, 1994; Walboomers *et al*, 1999; Richman *et al*, 2002**).** In malignant transformation of uterine cervical epithelia, integration of high-risk HPV DNA into the host genome is considered an important step (zur Hausen, 1991). Viral DNA integration leads to disruption of the HPV 16 *E2* gene (Kalantari *et al*, 1998), which is a negative regulator of the *E6/E7* promoter. Consequently, the *E2* disruption leads to an increased expression of E6 and E7 viral oncoproteins that target the p53 and retinoblastoma (pRb) tumour suppressor proteins, respectively, and downregulates their antitumour functions (zur Hausen, 1991; Romanczuk and Howley, 1992).

A number of studies reported HPV DNA detection in extragenital cancers as well, although the aetiological involvement of HPV in those malignancies is still controversial (Snijders *et al*, 1997; Gillison and Shah, 2003; Castillo *et al*, 2006). The association between HPV and oesophageal squamous cell carcinoma (ESCC) was first reported by Syrjanen in 1982. Since then, HPV infection has received attention as a possible risk factor for ESCC development (Syrjanen, 1982**).** An extensive review by Syrjanen published in 2002 showed that HPV was positive in 22.9% of 1485 ESCCs analysed by *in situ* hybridisation (ISH) and in 15.2% of 2020 ESCCs analysed by PCR (Syrjanen, 2002). Accumulating evidence on the presence of HPV DNA in normal oesophageal epithelium (Chang *et al*, 2000; Zhou *et al*, 2003) and in cancer precursor lesions (Pillai and Nair, 2000; Astori *et al*, 2001) suggests an involvement of HPV in very early stages of the classical dysplasia–carcinoma sequence. The notion was also supported by the demonstration of HPV infection using serologic assays with enzyme-linked immunosorbent assays (ELISAs) (Han *et al*, 1996).

HPV DNA detection rates in ESCC samples appear to be different from area to area. An explanation, offered by Syrjanen in his review, is the hypothesis that the contribution of oncogenic HPVs to ESCC risk is higher in the areas with high ESCC risks (Syrjanen, 1982). Indeed, recent studies conducted in areas with a high ESCC risk showed fairly high detection rates of high-risk HPV DNA in cancer specimens (Chang *et al*, 2000; Shen *et al*, 2002; Farhadi *et al*, 2005). His hypothesis was also supported by a recent study conducted in Anyang area in China. The study showed that the detection rate of HPV, particularly high-risk genotypes, in specimens collected by balloon cytology examinations appeared to be higher in a village with a relatively high ESCC incidence when compared to a village with a low ESCC risk (Li *et al*, 2001). However, the finding could not be confirmed by a study conducted among high-ESCC risk population in Linxian, China. In this study, the infection of oesophageal cells with high-risk HPV genotypes occurs in 13% of asymptomatic adults with no evidence of squamous dysplasia and a similar proportion of individuals with mild, moderate or severe dysplasia, suggesting that HPV infection is not a major risk factor for ESCC in this high-risk Chinese population (Gao *et al*, 2006). On top of that, an ESCC case–control study nested in a cohort in Linxian, China, could not find any evident case–control differences in the levels of serum antibody against HPV 16, 18 or 73 (Kamangar *et al*, 2006). Although the studies in Linxian could not confirm the relationship between HPV and ESCC, the fact does not necessarily deny the presence of such a relationship in other areas as the risk factor of ESCC is known to be heterogeneous. In fact, nutritional factors were the most important aetiological component of ESCC in Linxian (Taylor *et al*, 2003**).**

In the present study, we compared HPV DNA detection rates in Gansu and Shandong, China, where age-adjusted mortality rates of oesophageal cancer were 29 out of 100 000 in 1996–2000 (Luo *et al*, 2002), and four out of 100 000 in 1973–1975 (Junshi *et al*, 1991), respectively. In HPV DNA detection, we used various techniques including PCR with the sensitive short PCR fragment (SPF10) primers (Ketler *et al*, 1999) and Southern blot hybridisation. In addition, we examined HPV integration into the cancer cell genome using real-time PCR, and expression of p53 and p16^INK4a^ in order to shed light on the aetiological roles of HPV in ESCC development.

## SUBJECTS AND METHODS

A total of 59 paraffin-embedded tissue samples of ESCC diagnosed during the period between 1994 and 2005 were obtained; 26 cases from Gansu, a province located in the northwestern part of China with a high incidence of ESCC, and 33 cases from Shandong, a province in the east coast of China with a low incidence of ESCC. Institutional Review Board of Kagoshima University Graduate School of Medical and Dental Sciences, Japan, approved the present study.

### DNA extraction

Formalin-fixed, paraffin-embedded samples were cut into 10 *μ*m slices and prepared according to the method described before (Greer *et al*, 1995), followed by digestion with proteinase K (200 *μ*g ml^−1^) at 55°C overnight. The presence of DNA was confirmed by PCR with *β*-globin (110 bp) using PCO3 primer 5′-ACACAACTGTGTTCACTAGC-3′ and PCO4 primer 5′-CAACTTCATCCACGTTCACC-3′.

### PCR amplification

The presence of HPV DNA was evaluated by PCR using GP5+/GP6+ primers (150 bp), consensus primers for the HPV *L1* gene (De Roda Husman *et al*, 1995). The PCR reaction was performed in a total volume of 25 *μ*l containing 1 U of HotStar Taq (QIAGEN, Hilden, Germany), 50 mM of each primer, 0.2 mM of each dNTP and 10 × HotStar Taq PCR buffer as supplied by the enzyme manufacturer (QIAGEN) (contains 1.5 mM MgCl_2_, Tris–Cl, KCl, (NH_4_)_2_SO_4_ pH 8.7). Different amounts of template for each sample were used (2.5 *μ*l, 5 *μ*l and 10 *μ*l). The amplification was carried out with initial enzyme activation at 95°C for 15 min, followed by 45 cycles that included a 1 min denaturation step at 94°C, a 2 min annealing step at 40°C and a 1.5 min chain elongation step at 72°C; and a final elongation at 72°C for 5 min. As positive control for amplification, full genomes of HPV 6 and 18 (kindly donated by Professor Harald zur Hausen, German Cancer Research Centre, Heidelberg, Germany) were used, and water as template was used as negative control. PCR products were visualised on 2% agarose gel with ethidium bromide staining by electrophoresis.

### Southern blot hybridisation

DNA from agarose gel was transferred by upward capillary blotting to a Hybond N+ nylon membrane (Amersham, Little Chalfont, UK) using 0.4 M NaOH buffer. Hybridisation and detection of HPV DNA was carried out using the ECL Nucleic Acid labelling and detection kit (Amersham, UK). The probe used to detect HPV DNA was obtained by purifying from agarose gel the GP5+/GP6+ PCR products from cloned HPV 6 and 18 using QIAEX II Extraction kit (QIAGEN, Germany).

### The INNO-LiPA genotyping system

A 65 bp region of *L1* gene of the HPV genome was amplified by PCR using SPF10 biotinylated primers 5′-GCiCAGGGiCACAATAATGG-3′ and 5′-GTiGTATCiACAACAGTAACAAA-3′, where ‘i’ indicates inosine (Ketler *et al*, 1999). The PCR products were visualised on 4% agarose gel with ethidium bromide staining by electrophoresis, and 10 *μ*l of this PCR product was denaturated and hybridised with specific oligonucleotide probes (25 HPV type-specific probes) immobilised as parallel lines on a nitrocellulose membrane strips, following the manufacturer's instructions (INNO-LiPA HPV genotyping kit, Innogenetics, Ghent, Belgium). The 28 probes for 25 different HPV genotypes in each INNO-LiPA strip are described elsewhere (Ketler *et al*, 1999). The strips were interpreted with a labelled acetate overlay with lines indicating the position of each probe relative to the reference mark.

### Real-time PCR

Real-time PCR was performed with the ABI Prism 7000 Sequence Detection System and SYBR-Green PCR master mix (PE, Applied Biosystems, Foster, CA, USA). The amplification conditions were 10 min at 95°C and a two-step cycle of 95°C for 15 s and 60°C for 60 s for a total of 45 cycles. The used primer sets were as follows: *E6F*: GAGAAACTGCAATGTTTCAGGACC; *E6*R: TGTATAGTTGTTTGCAGCTCTGTGC; *E2*F: AACGAAGTATCCTCTCCTGAAATTATTAG and *E2*R: CCAAGGCGACGGCTTTG.

Those primers amplify a fragment of *E6* (81 bp) and *E2* (76 bp) ORFs, respectively (Peitsaro *et al*, 2002). The final concentration of primers was 0.5 *μ*M. Two standard curves for *E2* and *E6* fragments were made by amplification of dilutions between 1 × 10^7^ and 1 × 10^1^ copies of HPV 16 cloned in pUC plasmid (kindly given by Dr Massimo Tommasino, IARC, Lyon, France). There was a linear relationship between the threshold cycle values plotted against the log of the copy number over the entire range of dilutions. All the experiments were made in duplicate. The specificity of amplification was confirmed using dissociation analysis starting at 60°C and agarose gel electrophoresis of amplified products.

### p16^INK4a^, p53 and E6 HPV 16 and 18 protein immunohistochemical staining (IHC)

Sections of a paraffin-embedded block with the thickness of 2–3 *μ*m were placed on silane-coated glass slides, and deparaffinised by passage through xylene. After the endogenous peroxidase activity was blocked with 0.3% H_2_O_2_/methanol, the slides were rehydrated with distilled water. For antigen retrieval, the slides were heated in 0.01 mol l^−1^ citrate buffer, pH 6.0, at 95°C for 5 min (at 121°C for p53 and E6) and then left to cool at room temperature (RT). After rinsing in 0.01 mol l^−1^ phosphate-buffered saline (PBS), pH 7.4, nonspecific antibody binding was reduced by incubating the sections with 1% foetal bovine serum in PBS at RT for 30 min. Then, the sections were incubated overnight at RT with a mouse monoclonal antibody against p16^INK4a^ protein (1 : 200 dilution, GST-p16^INK4^, PharMingen International, San Diego, CA, USA), p53 protein (1 : 50 dilution, DO-7, Dako Japan Co., Ltd, Kyoto, Japan) or HPV 16 E6/18 E6 (1 : 50 dilution, SC-460, Santa Cruz Biotechnology, Santa Cruz, CA, USA). After washing thoroughly with PBS, the slides were incubated with biotinylated horse anti-mouse IgG (1 : 200 dilution) for 30 min, washed with PBS followed by a 1 : 50 dilution (1 : 100 dilution for p53 and E6) of the avidin–biotin–peroxidase complex (Vectastain elite ABC kit, Vector Laboratories, Burlingame, CA, USA) for an additional 30 min and washed with PBS. The peroxidase signal was visualised by treatment with DAB substrate-chromogen system (DAKO, Japan) for 10 min. Finally, the sections were stained lightly with haematoxylin. In statistical analysis, the cases with less than 10% cells stained positive were classified as negative cases, and the other cases were regarded as positive cases (Xu *et al*, 2004).

### Statistical analysis

Fisher's exact test was conducted using STATA version 8. The association between the presence of HPV 16 genome and tumour differentiation was also examined using area as a covariate in a multivariate logistic model by LogXact version 2.1. Tumour differentiation was used as a binomial variable: 0=poorly or moderately differentiated and 1=well differentiated. All the *P*-values presented are two sided.

## RESULTS

Table 1 summarises the clinicopathlogical features of ESCC cases examined in the present study. The age of the patients ranged from 27 to 83 years with a mean of 61±10 years. Male and female cases numbered 48 (81%) and 11 (19%), respectively.

First, we examined all the 59 samples using GP5+/GP6+ primers for PCR and visualised only five HPV-positive cases in agarose gel. When agarose-gel electrophoresis was replaced with Southern blot hybridisation, HPV DNA was detected in an additional 11 cases (in total 16 cases). As an alternative approach, we examined cancer specimens using SPF10 primers, and amplicons were visualised on 4% agarose gel. In this method, HPV genome was confirmed to be present in all the 16 HPV-positive samples identified by Southern blot hybridisation. In addition, three cases became HPV positive in this new approach, and, in total, we detected HPV DNA in 19 specimens, 17 samples from Gansu (65%) and only two samples from Shandong (6%). The difference in detection rates between the two regions was highly significant (*P*<0.001, Fisher's exact test). In the following, we present the results using SPF10 primers.

Table 2 shows the detection rate of HPV according to clinicopathological characteristics. HPV detection rate did not show any statistically significant association with sex or age. ESCCs from Shandong tended to be well differentiated, and Gansu cases were predominated by moderately differentiated tumours. There was no evidence indicating the association of high-risk HPV presence with the expressions of p53 or p16^INK4a^.

We could not detect HPV 16/18 E6 protein expression by IHC in any of the HPV-16-positive samples.

**Figure 1:**
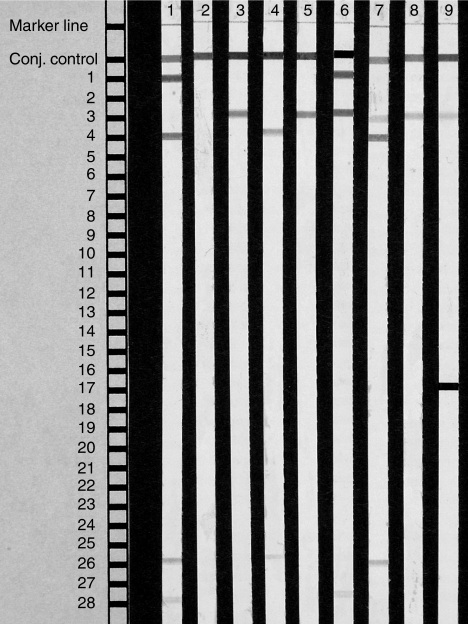
Representative examples of INNO-LiPA HPV genotyping assay. Strip 1: positive control of HPV types 6 and 18; strip 2: negative control; strips 3–9 are ESCC samples; strip 3: HPV 16; strip 4: HPV 18; strip 5: HPV 16; strip 6: HPV 6 and 16; strip 7: HPV 16 and 18; strip 8: HPV 16 and strip 9: HPV 16 and 51.

Table 3 presents the HPV genotype identified by INNO-LiPA HPV genotyping assay, where a single-strip analysis can identify as many as 25 different HPV genotypes (Figure 1). The most prevalent genotype was HPV 16, detected in 15 samples (79%), followed by HPV 18, found in three samples (16%). We found more than one HPV genotypes in three (16%) of the genotyped samples. The coinfection of two high-risk HPV types, HPV 16/18 and HPV 16/51, were found in poorly differentiated tumours, while the coinfection of one low- and one high-risk HPV (HPV 6/16) was shown in a well-differentiated tumour. All the cases with coinfection were male. The other factors, including age and the expression of p16^INK4a^ and p53, showed no significant associations with multiple HPV genotype detection.

Since the majority of HPV genotype detected was HPV 16, we compared HPV-16-positive and HPV-negative tumours with respect to clinicopathological features (data not shown). The area difference was statistically significant (*P*<0.001, Fisher's exact test). The presence of HPV 16 genome was more frequent in moderately and poorly differentiated carcinomas. The observed association was statistically significant (*P*=0.011, Fisher's exact test), but was not significant in a multivariate analysis adjusting for area (*P*=0.813).

Table 3 also summarises the results of real-time PCR analysis. The quantity of HPV 16 *E6* DNA ranged from 0.001 to 283.29 copies ng^−1^ of genomic DNA. The results from repeated analyses showed a difference less than 5% in all the cases except one, which showed difference as large as 18%. Viral load was not related to any clinicopathological features listed in Table 1 (data not shown). *E2/E6* ratio was determined by real-time PCR. In nine (60%) cases, *E2* DNA was not detected. In the rest of the cases, *E2* DNA was detected, but the *E2/E6* ratio was less than unity.

## DISCUSSION

In the present study, using PCR with SPF10 primers or PCR with GP5+/GP6+ primers combined with Southern blot hybridisation, we detected HPV DNA in 6% of Shandong samples while HPV DNA was positive in 65% of samples from Gansu, where ESCC incidence is much higher than in Shandong. Our finding suggests possibility of a strong geographical difference in the proportion of HPV-associated ESCCs. Note, however, that our ESCC cases were convenient samples and, therefore, may not represent the ESCC cases in the study areas. All HPV strains that could be genotyped were found to be high-risk types. Prevalent HPV genotypes were HPV 16 and 18, which were found in 79 and 16% of ESCC samples, respectively.

Detection rate varied upon the method used, with a high sensitivity when using Southern blot hybridisation, and it was even higher when using SPF10 primers PCR. However, even if such a sensitive detection method was used, HPV genome was detectable only in a small portion of cancer specimens collected from Shandong (6%).

Using real-time PCR, we confirmed the presence of HPV 16 in 15 ESCC samples. Since HPV integration is considered to result in deletion of the *E2* gene (Jeon and Lambert, 1995), we determined the status of HPV in the host cells on the basis of the *E2/E6* ratio (Peitsaro *et al*, 2002). When the ratio was equal to or higher than unity, all the HPV genome was considered to be in an episomal form and not integrated. On the other hand, the lack of amplified HPV *E2* genome was considered to indicate the integration of all HPV genome into the host genome. When the *E2/E6* ratio was larger than zero and smaller than unity, we considered the condition as the mixture of episomal and integrated forms, where a portion of HPV genome was integrated into the host genome. In the present study, the integrated form of HPV was detected in all the HPV-positive specimens, including 60% of the samples without detectable *E2* DNA. We compared those findings with those of cervical cancers. Using 12 cervical cancer samples positive for HPV 16 (unpublished data), we determined *E2/E6* ratio and found that 92% of the samples had a mixture of episomal and integrated forms of HPV genome, and 8% of the samples had HPV integrated into the host genome. The method, similar to ours, showed that 65–97% of the cervical cancer cases had HPV 16 integration, which was frequently accompanied by episomal-form HPV (Peitsaro *et al*, 2002; Arias-Pulido *et al*, 2006**).**

In the present study, a viral load in each specimen was expressed as the number of HPV copies/genome equivalent or cell, using a conversion factor of 6.6 pg of DNA cell^−1^ in order to make comparisons with the results of other studies (Si *et al*, 2003). Real-time PCR analysis showed a very low viral load, ranging from 0.001 to 283.29 copies ng^−1^ of genomic DNA, which corresponds to <1–2 copies cell^−1^. The copy numbers of HPV 16 as low as what was observed in the present study were also reported by a Chinese study on oesophageal cancer tissues (<1 to 157 copies cell^−1^) (Si *et al*, 2003), and by a Finnish study on head and neck SCCs (4.6 to 49 copies cell^−1^) (Koskinen *et al*, 2003**).** Considering the presence of relatively low viral loads of HPV DNA in some cervical carcinoma cell lines, for example SiHa (1–2 copies of HPV 16 cell^−1^) and HeLa (10–50 copies of HPV 18 cell^−1^), the low viral load in ESCC may be sufficient to promote carcinogenesis, especially if viral DNA is integrated into the cell (Si *et al*, 2005). The integration of HPV DNA into the host genome, rather than the mere presence of the episomal HPV DNA, may contribute to oesophageal carcinogenesis.

A serological study in Linxian, China, where the ESCC risk is extremely high (Kamangar *et al*, 2006), found no significant association between seropositivity of HPV 16 and ESCC risk, although serum antibodies against HPV antigens are considered good markers of exposure to HPV in the past. Their results can be explained by the decrease or loss of antibodies against HPV capsid antigens during several decades after HPV infection until study subjects were examined and their blood samples were drawn (Lagergren *et al*, 1999). The viral load of HPV may also decrease over those years and the viral-load decrease may also take place during the process of cancer development. Therefore, the low viral load and no expression of HPV 16 E6 protein in the present study may be explained by the decrease of HPV viral load after infection and/or a ‘hit and run’ mechanism, which has been proposed for the association between bovine papillomavirus type 4 and oesophageal cancer in cattle (Campo, 1987).

HPV E6 and E7 are considered to be the major oncoprotein interfering with cell cycle regulation. The E6 protein of high-risk HPV binds to p53, leading to rapid degradation of p53 through the ubiquitin pathway (Scheffner *et al*, 1990). On the other hand, the E7 protein binds and phosphorylates the tumour suppressor pRb and inhibits its binding to E2F. Released E2F transcriptional factor stimulates p16 transcription, leading to p16^INK4a^ overexpression, which is also caused by the loss of the negative feedback from free pRb, disrupted by HPV E7 (Scheffner *et al*, 1990; Lukas *et al*, 1995). Indeed, overexpression of p16^INK4a^ is known to be observed in cancers of the uterine cervix (Schorge *et al*, 2004), which is almost always associated with HPV infection. In order to shed light on the aetiological involvement of HPV, we conducted IHC of p53 and p16^INK4a^. However, we could not find any evidence indicating the association of high-risk HPV presence with the expressions of p53 or p16^INK4a^. Note, however, that p16^INK4a^ is frequently inactivated through hypermethylation of p16^INK4a^ promoter and through deletion at near-p16 loci (Serrano *et al*, 1993; Xing *et al*, 1999; Tokunaga *et al*, 2002**).** In our study, we found a downregulation of p16^INK4a^ expression in 68% of ESCC cases regardless of HPV presence. The failure to detect p16^INK4a^ overexpression in HPV-positive ESCC may therefore be explained by the fact that p16 is downregulated through hypermethylation.

It should be noted that the cancer specimens used in the present study contain noncancerous tissues, and that our findings do not necessarily indicate the presence of HPV in carcinoma cells. Studies showed the presence of HPV 16 E6 protein in the cytoplasm and nucleus of ESCCs, which showed the presence of HPV 16 in PCR analysis as well (Li *et al*, 2001; Yao *et al*, 2006**).** In the present study, we could not detect viral E6 protein expression by IHC in any of the HPV-16-positive samples.

Studies on the presence of HPV DNA in cervical samples showed that 10% or more of clinical lesions contain at least two different HPV genotypes (Rousseau *et al*, 2003; Schellekens *et al*, 2003**).** In the present study, we observed multiple infection of HPV with different genotypes in 16% of the genotyped samples, using INNO-LiPA genotyping assay. Double infections in ESCC have also been reported by a study in China (Lavergne and De Villiers, 1999). Interestingly, in our study, the ESCC sample with double infection of a high- and a low-risk HPV type (16/6) had the least number of viral copies (0.001 copies ng^−1^). It is of note that Silins *et al*. (1999) suggested that infection with HPV 6 interfere with HPV 16 in cervical carcinogenesis. Further studies on these interactions are warranted as HPV coinfections in ESCC may affect the HPV life cycle as well as disease progression as suggested by McLaughlin-Drubin and Meyers (2004).

In conclusion, the present study showed that a large proportion of ESCC specimens harbour HPV 16 genome in the integrated form in a certain area with a high ESCC incidence. Detection rate varied upon the method used, with a higher sensitivity when using Southern blot hybridisation or SPF10 primers PCR. Real-time PCR analysis suggested the presence of only a small number of HPV 16 copies in carcinoma cells. There was no HPV 16/18 E6 protein expression, and on top of that, HPV 16 presence was not related to the expression of p16^INK4a^ or p53 protein. Further studies seem warranted to examine the possible aetiological roles of HPV in ESCC.

## Figures and Tables

**Table 1:** Clinicopathological features of ESCC cases in this study

	**Shandong**	**Gansu**	
	***N* (%)**	***N* (%)**	***P***-value^*^
Total	33 (100)	26 (100)	
			
*Gender*			0.740
Male	26 (79)	22 (85)	
Female	7 (21)	4 (15)	
			
*Age (years)* a			0.562
<55	10 (31)	5 (19)	
55–64	10 (31)	8 (31)	
⩾65	12 (38)	13 (50)	
			
*Differentiation grade*			<0.001
Well	20 (61)	2 (8)	
Moderate	8 (24)	14 (54)	
Poor	5 (15)	10 (38)	
			
*p16^INK4a^ protein expression (%)*			0.303
<10	23 (70)	17 (65)	
10–79	3 (9)	6 (23)	
⩾80	7 (21)	3 (12)	
			
*p53 protein expression (%)*			0.064
<10	19 (58)	9 (35)	
10–79	7 (21)	4 (15)	
⩾80	7 (21)	13 (50)	

^*^P-values were obtained by Fisher's exact test.

a Information on age was missing in a case.

**Table 2:** HPV DNA detection in ESCC by PCR using SPF10 primers

	**All cases**	**HPV positive**	**HPV negative**	
	***N* (%)**	***N* (%)**	***N* (%)**	***P***-value^*^
All	59 (100%)	19 (32%)	40 (68%)	
				
*Region*				<0.001
Shandong	33 (100)	2 (6)	31 (94)	
Gansu	26 (100)	17 (65)	9 (35)	
				
*Gender*				1.000
Male	48 (100)	16 (33)	32 (67)	
Female	11 (100)	3 (33)	8 (67)	
				
*Age (years)* a				0.881
<55	15 (100)	4 (27)	11 (73)	
55–64	18 (100)	6 (33)	12 (67)	
⩾65	25 (100)	9 (36)	16 (64)	
				
*Differentiation grade*				0.008
Well	22 (100)	2 (9)	20 (91)	
Moderate	22 (100)	9 (41)	13 (59)	
Poor	15 (100)	8 (53)	7 (47)	
				
*p16^INK4a^ protein expression (%)*				0.160
<10	40 (100)	16 (40)	24 (60)	
10–79	9 (100)	2 (22)	7 (78)	
⩾80	10 (100)	1 (10)	9 (90)	
				
*p53 protein expression (%)*				0.813
<10	28 (100)	8 (29)	20 (71)	
10–79	11 (100)	4 (36)	7 (64)	
⩾80	20 (100)	7 (35)	13 (65)	

^*^P-values were obtained by Fisher's exact test.

a Information on age was missing in a case.

**Table 3:** HPV genotypes by INNO-LiPA and HPV 16 viral load and physical status by real-time PCR in ESCC

**Sample**	**Area**	**Genotype**	***E6* copies/ng**	***E2* copies/ng**	***E2/E6***	**Status**
1	Gansu	16	40.648	6.516	0.160	Mix
2	Gansu	16	0.019	0.007	0.372	Mix
3	Gansu	16	6.537	0.001	0.000	Integrated
4	Gansu	16	0.366	0.000	0.000	Integrated
5	Gansu	16	7.200	0.000	0.000	Integrated
6	Gansu	16	49.516	2.603	0.053	Mix
7^a^	Gansu	16	11.242	0.074	0.007	Mix
8	Gansu	16	0.436	0.000	0.000	Integrated
9	Gansu	16	283.293	20.908	0.074	Mix
10	Gansu	16	0.374	0.000	0.000	Integrated
11^b^	Gansu	16	5.696	0.000	0.000	Integrated
12	Gansu	16	0.351	0.005	0.015	Mix
13	Gansu	16	1.272	0.000	0.000	Integrated
14	Gansu	16	0.656	0.000	0.000	Integrated
15	Gansu	18	ND^c^	ND	ND	ND
16	Gansu	Undetermined	ND	ND	ND	ND
17	Gansu	Undetermined	ND	ND	ND	ND
18^d^	Shandong	16	0.001	0.000	0.000	Integrated
19	Shandong	18	ND	ND	ND	ND

a Sample with HPV 16/18 coinfection.

b Sample with HPV 16/51 coinfection.

c Not done.

d Sample with HPV 6/16 coinfection.
